# Declining Rates of Tobacco Use in the Japanese Medical Profession, 1965–2009

**DOI:** 10.2188/jea.JE20120121

**Published:** 2013-01-05

**Authors:** Derek R. Smith, Koji Wada

**Affiliations:** 1 School of Health Sciences, Faculty of Health, University of Newcastle, Ourimbah, Australia; 2 Department of Public Health, Kitasato University School of Medicine, Sagamihara, Kanagawa, Japan

**Keywords:** smoking, tobacco, physician, doctor, Japan

## Abstract

**Background:**

Although there has been a downward trend in smoking rates among medical doctors in recent years, rates have been higher among Japanese doctors when compared internationally.

**Methods:**

We extensively reviewed all published English- and Japanese-language articles that reported the smoking rates of Japanese doctors.

**Results:**

A total of 36 articles were examined, most of which had been conducted as postal surveys, usually by a national, prefectural, or local medical association. Sample sizes ranged from 17 to 11 773, and response rates ranged from 33% to 91%. National surveys conducted between 1965 and 2009 suggest that there has been a statistically significant (*P* < 0.0001) decline in smoking rates among Japanese doctors (from around 68% to 16% among males and from 19% to 5% among females).

**Conclusions:**

Overall, the published data reveal a significant decline in smoking rates among Japanese doctors since 1965, especially among men. Although less than one-fifth of Japanese male doctors now smoke, more work needs to be done in tobacco control to help further reduce the burden of smoking, especially in medical schools.

## INTRODUCTION

At least 5 million people die each year from the effects of tobacco use, and current projections suggest that 8 million smokers will be dying from smoking every year by 2030. There are over 1 billion smokers in the world today, and up to half of them will die prematurely from a tobacco-related disease.^1^ However, this need not be so. Among the top 5 risk factors for mortality, tobacco use is the most preventable. Therefore, because they are on the front lines of primary care, health professionals have a key role in tobacco control at both the individual and societal levels. In addition, their professional organizations can show leadership and influence local, national, and global efforts to combat the current epidemic.^2^ Given these clear responsibilities as providers of care and public role models, it is surprising that some healthcare professionals continue to smoke. As a result, tobacco use among this group can be seen as one of the last “unmet challenges” for those working in public health.^3^

As one of the most well-respected health professionals, doctors have a major role in tobacco control, particularly as exemplars and role models of healthy lifestyles. Doctors who smoke naturally attract public scepticism,^4^ and for at least 35 years it has been suggested that doctors could best persuade their patients to quit tobacco use if they themselves did not smoke.^5^^,^^6^ For these reasons, medical professionals tend to give up smoking earlier than the general population in which they operate.^7^ Substantial progress has been made in reducing the smoking rates of doctors in many countries,^7^ although these gains have not necessarily been uniform or sustained. For example, a large review of the topic, published in 1989,^8^ reported a downward trend in smoking rates among male doctors in most countries; however, the decrease was less obvious among women. On the other hand, a 2007 review^9^ of tobacco use in the medical profession found that doctors in some developing countries and newly developing regions still smoked at relatively high rates. Among the industrialized countries examined, Japanese doctors have relatively high smoking rates.^9^ Approximately 290 000 doctors currently practice in Japan.^10^ Despite this fact, there has been no detailed examination of smoking rates among Japanese doctors. Moreover, recent trends in tobacco use in this profession have not been comprehensively investigated. Thus, we conducted a detailed review of published reports that had data on smoking rates among Japanese doctors, and examined smoking trends within this group over time.

## METHODS

The first stage of our literature review was undertaken in the first half of 2012 and involved an extensive English-language search using the PubMed website of the US National Library of Medicine (NLM).^11^ The search terms included: tobacco, smoke, physician, doctor, medical, and Japan. Various keyword variations and combinations were used, as were the Boolean operators AND, OR, and *, to identify keyword variations such as Japan and Japanese, and smoke and smoking. A Japanese-language search was then undertaken using the Japana Centra Revuo Medicina’s *Ichushi* Website.^12^

Japanese-language sources identified during this search included the following journals (with English-language titles in parentheses): *Igaku no Ayumi* (Journal of Clinical and Experimental Medicine), *Kanagawa Igakukai Zasshi* (Journal of the Kanagawa Medical Association), *Kawasaki Igakukai Shi* (Kawasaki Medical Journal), *Kikanshi Gaku* (Journal of the Japan Society for Bronchology), *Kousei no Shihyo* (Journal of Health and Welfare Statistics), *Nihon Kokyu Kanri Gakkaishi* (Journal of Japan Society for Respiratory Care), *Nihon Koshu Eisei Zasshi* (Japanese Journal of Public Health), *Nihon Kyobu Shikkan Gakkai Zasshi* (Japanese Journal of Thoracic Diseases), *Nippon Eiseigaku Zasshi* (Japanese Journal of Hygiene), Nippon Iji Shinpo (Japan Medical Journal), *Nippon Ishikai Zasshi* (Journal of the Japan Medical Association), *Osakufu Ishikai News* (Osaka Medical Association News), *Toukeibugan* (Japanese Journal of Head and Neck Cancer), and the *Toyama Kenritsu Chuo Byoin Igaku Zasshi* (Medical Journal of the Toyama Prefectural Central Hospital).

Full text copies of all articles were obtained and examined in detail, and the reference lists were then reviewed to locate additional sources, many of which did not appear to have been indexed by PubMed or *Ichushi*. All data were compiled into 2 tables (one for English articles and one for Japanese articles), which were then sorted by the year the study was conducted (from earliest to most recent). When the year of the study was not specified, the publication year was used. The earliest study was conducted in 1965, and the most recent was undertaken in 2009; thus, the period 1965–2009 was selected for the current review. Smoking prevalence rates and study response rates for each article were then rounded to the nearest whole number and included in the tables. All data were added to a spreadsheet program and analyzed using statistical techniques, including mean values and linear regression for trend.^13^

## RESULTS AND DISCUSSION

A total of 20 English reports describing 17 studies of the smoking habits of Japanese doctors were published between 1967 and 2011, as shown in Table 1. Most of the English studies (75%) were postal surveys, and had usually been conducted by a national, prefectural, or local medical association—the Japan Medical Association (JMA) was the most common surveying body (accounting for 32%). A total of 17 Japanese reports describing 19 different studies were identified for the period 1980–2010, as shown in Table 2. Most (58%) were postal surveys, and had often been conducted by medical associations or specialist groups; 26% were conducted at single hospitals. Four studies had used data from surveys of JMA members. An examination of all studies (published in both languages) showed that the prevalence of smoking among Japanese doctors ranged from 7% to 66% between 1965 and 2009 (7%–68% among men and 0%–19% among women).

**Table 1.** Research publications describing the smoking rates of Japanese doctors (published in English)

Location	Data Source	Year^b^	Method	Current Smoker^a^			Ex- Smoker	Never Smoker	Study Details		Authors
	
				All	Male	Female			Sample Size	Response Rate^c^	
Various^d^	Japan Medical Association	1965	Postal Survey	66%	68%	19%	17%	17%	11 773	49%	Nishizumi & Kuratsune, 1967^14^

Fukuoka	Fukuoka Prefectural Medical Association	1983	Postal Survey	42%	43%	9%	—	—	4190	84%	Kaetsu et al, 2002^52^

Nationwide	Pharmaceutical company^e^	1986*	n/s	39%	—	—	—	—	9456	—	Kawane, 1986^53^

Nationwide	Japan Society of Chest Diseases	1989	Postal Survey	25%	26%	6%	39%	36%	3640	59%	Kawane, 1991,^54^ 1993,^55^ 2001^56^

Fukuoka	Fukuoka Prefectural Medical Association	1990	Postal Survey	32%	33%	5%	—	—	3565	63%	Kaetsu et al, 2002^57^

Toyama	Cardiologists at 3 University Hospitals	1992	Hand-delivered	40%	—	—	—	—	17	—	Miwa et al, 1995^58^

Nationwide	World Health Organization^e^	1994*	n/s	44%	—	—	—	—	—	—	Audet, 1994^59^

Kanagawa	Kawasaki Medical School Hospital	1994	Hand-delivered	29%	—	—	21%	50%	163	60%	Kawane & Soejima, 1996^60^

Tokyo	A Central Tokyo Hospital^f^	1994	Postal Survey	21%	24%	7%	47%	31%	323	71%	Kawakami et al, 1997^61^

Fukui	Fukui Prefectural Medical Association	1996–97	Postal Survey	26%	28%	5%	46%	28%	709	91%	Kawahara et al, 2000^62^

Various^g^	Japan Medical Association	2000	Postal Survey	—	27%	7%	—	—	3771	84%	Ohida et al, 2001^63^^,^^64^

Various^g^	Japan Medical Association	2004	Postal Survey	16%	22%	5%	30%	—	3633	81%	Kaneita et al, 2008^65^

Fukui	Graduates from the University of Fukui	2004	Postal Survey	—	13%	4%	—	46%	261	48%	Kanayama et al, 2012^10^

Various^h^	Medical Residents in 16 Training Hospitals	2005	Postal Survey	19%	—	—	—	—	196	86%	Wada et al, 2007^66^

Nationwide	Surgeons and Anesthesiologists^i^	2005	Postal Survey	12%	—	—	30%	58%	1063	53%	Kai et al, 2008^67^

Various^g^	Japan Medical Association	2008	Postal Survey	—	15%	5%	—	—	3486	77%	Kaneita et al, 2010^68^

Nationwide	Japan Medical Association	2009	Postal Survey	14%	16%	5%	12%	74%	4055	41%	Wada et al, 2011^69^

^a^Smoking rates rounded to nearest whole number, ^b^Year study was conducted (when not specified, publication year is listed*), ^c^Response rates rounded to nearest whole number, ^d^Hyogo, Tottori, Shimane, Okayama, Hiroshima, Yamaguchi, Tokushima, Kagawa, Ehime, Kochi, Fukuoka, Saga, Nagasaki, Kumamoto, Oita, Miyazaki, and Kagoshima, ^e^Additional details of study were not specified, ^f^Location as stated in article, ^g^Hokkaido/Tohoku, Kanto, Chubu, Kinki, Tyugoku/Shikoku, and Kyushu, ^h^Hokkaido/Tohoku, Kanto, Chubu, Kansai, and Kyushu, ^i^Board-certified members of the Japanese Association for Thoracic Surgery or the Japanese Society of Anaesthesiologists, n/s = Not clearly specified.

**Table 2.** Research publications describing the smoking rates of Japanese doctors (published in Japanese)

Location	Specialty	Year^b^	Method	Current Smoker^a^			Ex- Smoker	Never Smoker	Study Details		Authors
	
				All	Male	Female			Sample Size	Response Rate^c^	
Osaka	Osaka Medical Association	1979	n/s	—	52%	12%	—	—	3197	58%	Yokota, 1980^70^

Saga	Saga Prefectural Medical Association	1983	Postal Survey	—	44%	—	42%	14%	729	81%	Nishizumi, 1986^71^

Osaka	Toyonaka City Medical Association	1987	Postal Survey	—	37%	8%	—	—	323	90%	Yokota, 1988^72^

Okayama	Kawasaki Medical School Hospital	1987	Hand-delivered	—	24%	—	33%	43%	120	50%	Kawane et al, 1989^73^

Kanagawa	Yokosuka City Hospital^d^	1988	Hand-delivered	40%	—	—	40%	20%	25	46%	Nomura et al, 1991^74^

Toyama	11 Medical Associations in Toyama	1989*	n/s	29%	—	—	—	—	1002	n/s	Igarashi & Muroya, 1989^75^

Nationwide	Japan Society of Chest Diseases	1989	Postal Survey	25%	26%	6%	39%	36%	3640	59%	Kawane & Soejima, 1991^76^

Kanagawa	Yokosuka City Hospital^d^	1990	Hand-delivered	44%	—	—	32%	24%	25	46%	Nomura et al, 1991^74^

Nationwide	Japan Society of Chest Diseases	1996	Conference Survey	23%	—	—	—	—	2411	65%	Kobayashi & Kitamura, 1997^77^

Gifu	Gifu Medical Hospital	1996	Hand-delivered	25%	25%	0%	22%	—	270	85%	Kano et al, 1999^78^

Fukui	Fukui Prefectural Medical Association	1996–7	Postal Survey	—	28%	8%	—	—	794	91%	Ohida et al, 2000^79^

Tokyo	Clinic-based Doctors^e^	1998*	Postal Survey	21%	24%	8%	49%	29%	245	68%	Kawakami et al, 1998^80^

Tokyo	Hospital-based Doctors^e^	1998*	Postal Survey	19%	23%	0%	33%	47%	366	68%	Kawakami et al, 1998^80^

Nationwide	Japan Medical Association	2000	Postal Survey	—	27%	7%	—	—	3885	87%	Sakurai & Ohida, 2000^81^

Nationwide	Japan Society for Respiratory Care	2003	Conference Survey	—	9%	0%	—	—	171	n/s	Takiguchi, et al^82^

Nationwide	Japan Medical Association	2004	Postal Survey	—	22%	5%	—	—	3776	86%	Kaneita & Ohida, 2005^83^

Nationwide	Japan Medical Association	2008	Postal Survey	—	15%	5%	—	—	3561	80%	Kaneita et al, 2009^84^

Nationwide	Japan Society for Head and Neck Cancer	2008	Postal Survey	7%	7%	3%	39%	53%	974	33%	Miyahara et al, 2009^85^

Various	Japan Medical Association	2009	Postal Survey	—	16%	5%	—	—	4055	41%	Wada et al, 2010^17^

^a^Smoking rates rounded to nearest whole number, ^b^Year study was conducted (when not specified, publication year is listed*), ^c^Response rates rounded to nearest whole number, ^d^Studies done at same location but at 2 different times (1988 and 1990), ^e^Studies done at the same time but at 2 different locations (clinics and general hospitals), n/s = Not clearly specified.

**Figure 1.**
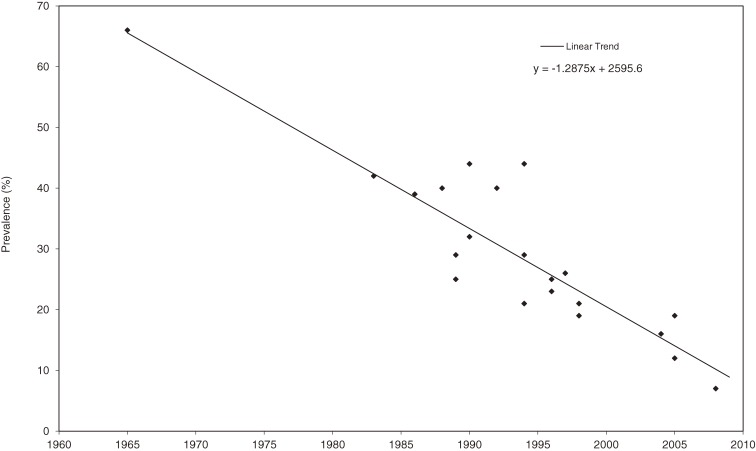
Declining overall trend in smoking among Japanese doctors, 1965–2009

**Figure 2.**
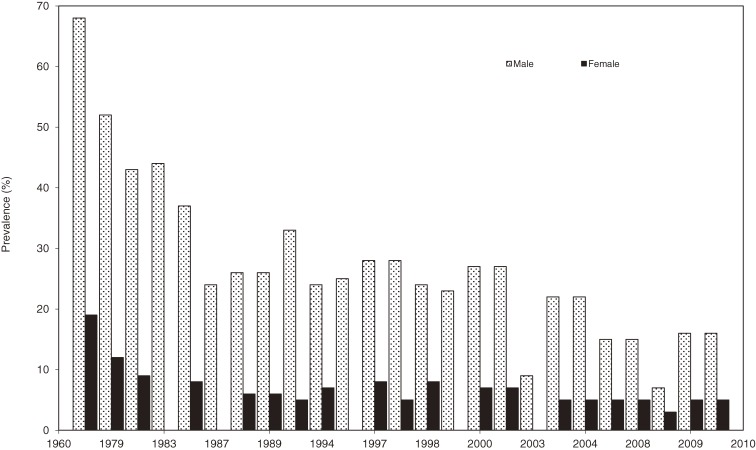
Declining prevalence of smoking among Japanese doctors by sex, 1965–2009

Sample sizes ranged from 17 to 11 773, and response rates ranged from 33% to 91%. Trend analysis of the published data showed a statistically significant decline in smoking rates among Japanese doctors since 1965 (*P* < 0.0001; y = −1.2875x + 2595.6) (Figure 1). Much of this decline was due to marked reductions in smoking rates among male doctors (*P* < 0.0001), from over 70% in the 1960s to around 10% in more-recent studies. A decline in tobacco use among female Japanese doctors also occurred over time, although the reduction was substantially less than that observed for men (from less than 20% in the 1960s to around 5% in recent studies) (Figure 2).

The earliest study of smoking rates among Japanese doctors appears to have been undertaken by Nishizumi and Kuratsune,^14^ whose 1967 article reported a smoking prevalence among Japanese doctors of 68% in men and 19% in women. In addition to providing groundbreaking national data on tobacco use among JMA members, their study proved to be a rich data source for later longitudinal investigations. In the 1980s, for example, Kono and colleagues published follow-up studies of doctors who had been enrolled in the original study.^15^^,^^16^ The most recent investigation of smoking among a national sample of Japanese doctors (again, JMA members) was published by Wada and colleagues in 2009^17^ and reported much reduced smoking rates of 16% for men and 5% for women. Similarly low smoking rates among female doctors, as compared with their male counterparts, have been recently reported in China.^18^ A plot of reported smoking rates from all studies (Figure 1) clearly demonstrates the magnitude of this decline in Japan, which is consistent with trends in many other countries. For example, the earliest large-scale reviews of doctors’ smoking habits appear to have been undertaken by Adriaanse and colleagues,^8^^,^^19^ who found that smoking prevalence rates had declined substantially in many countries since the 1950s. An examination of individual studies since then suggests that the most significant long-term declines in physician smoking rates have been in the United States,^20^ the United Kingdom,^21^ Australia,^22^ and New Zealand.^23^ Much of the decline in smoking rates among Japanese doctors has occurred among men, as shown in Figure 2.

Although smoking rates in the Japanese medical profession have clearly fallen, Japan remains somewhat of a paradox in tobacco control because it is a developed country with a relatively high societal smoking prevalence rate. In 1950, for example, the national smoking prevalence rate among Japanese citizens older than 19 years was around 82% in men and 13% in women.^24^ However, there has been a long-term decline in Japanese smoking rates since the 1970s,^13^ mostly due to control measures instigated by various government agencies, which had themselves been influenced by social movements.^25^ By 1993, the smoking rate among Japanese men had decreased to around 60%, but had increased to around 14% among women. Furthermore, between 1969 and 1996, the average daily cigarette consumption per Japanese smoker continuously increased.^24^ According to the World Health Organization (WHO), approximately 38% of Japanese men and 11% of Japanese women older than 20 years currently smoke.^26^ Currently, smoking has one of the largest impacts on life expectancy in Japan^27^^,^^28^ and causes a significant health burden, especially among men.^29^ This situation is worsened by the current low rate of cessation attempts by Japanese men.^30^

Doctors have an important role in the fight against smoking.^31^ Although there has been a clear decline in smoking rates among Japanese doctors since 1965, not all studies reported positive attitudes towards tobacco control. A 2001 study of Japanese university hospitals, for example, found that less than 2% of the surveyed facilities were aiming for complete prohibition of smoking on the hospital site.^32^ A research group on prospective measures for anti-smoking and passive smoking in Japan has now been funded by the Japanese Ministry of Health, Labour and Welfare, and its website shows current information on the status of Clean-Air Medical Universities in Japan.^33^ Interestingly, smoking rates among members of the Japanese Cancer Association (JCA) increased between 2004 and 2006 (from 6% to 9%),^34^ although it is important to remember that these figures are still far below community smoking rates in Japan. One confounder may be the next generation of health professionals, as research suggests that, despite the increasing proliferation of campus-wide smoking bans, too many Japanese university students are still using tobacco.^35^ A 2007 survey, for example, reported that dental students had the highest student smoking rate in Japan,^36^ while an international review of this topic also indicated that Japan has one of the higher smoking rates among dental students in industrialized countries.^37^ This may reflect higher smoking rates within the Japanese dental profession, as compared with their peers in other countries.^38^ Two recent studies of Japanese dentists reported that 25% to 28% were current smokers,^39^^,^^40^ while a national survey of Japan Dental Association (JDA) members documented a smoking prevalence of 30% among men and 11% among women.^41^

Smoking appears to be less common in the Japanese medical profession than in the dental profession; a trend that also includes medical students. A recent study in Nagoya, for example, documented a medical student smoking rate of only 6% among men and 2% among women.^42^ This is a decline from the 14% smoking prevalence rate documented among Japanese medical students between 2006 and 2007^43^ and is a major improvement from the rates reported in a variety of earlier studies.^44^ It is unclear whether Japanese medical students who smoke will continue to do so when they graduate and start work as doctors. Although focusing tobacco control education on doctors before they graduate may be an appropriate way forward, such efforts would clearly need to be part of a wider strategy that targets students in all health disciplines, especially dentistry. Enforcement of a total smoking ban on all university campuses may be a way forward, as this has recently been shown to be an effective strategy for increasing the quit rate among teachers in some Japanese primary and high schools.^45^

Despite these ongoing challenges, Japanese health care professionals have much to celebrate with regard to tobacco control. In recent times, monitoring the societal prevalence of smoking has become essential for understanding the tobacco epidemic, and Japan is one of the world’s highest-achieving countries in this respect.^46^ Japan has been a signatory to the WHO Framework Convention on Tobacco Control (FCTC) since 2004,^46^ and smoking rates among Japanese men declined by almost half between 1990 and 2009 (falling from 61% to 38%).^26^ Nishizumi and Kuratsune’s 1967 paper on smoking habits within the Japanese medical profession^14^ can be seen as an archetypal Asian epidemiologic study, all the more so given that it was published in English in a Japanese journal. Furthermore, it was a Japanese doctor and epidemiologist, Takeshi Hirayama,^47^ who conclusively proved the dangers of passive smoking in his groundbreaking 1981 study published in the British Medical Journal.^48^ Hirayama has since been described by the anti-tobacco advocate, Professor Judith Mackay, as the “grandfather of Asian epidemiology.”^49^ Much can still be learned from his work in public health epidemiology.

Various Japanese medical associations are exercising professional leadership in tobacco control. Since 2008, the largest of these, the JMA, has formed a group of 9 doctors, known as the Project Committee Concerning Health Support for JMA Members Working in Hospitals. The JMA has also released a 7-point declaration on tobacco control.^50^ Since 2003, the Japanese Respiratory Society has required those seeking board certification as respiratory physicians to be nonsmokers.^51^ The application of these measures, combined with the significant reductions in overall smoking rates observed since 1965, represents a clear move toward achieving a smoke-free medical workforce in Japan.

## CONCLUSION

The published data show a significant decline in smoking rates among Japanese doctors since 1965, especially among men. Although less than one-fifth of Japanese male doctors now smoke, more work in tobacco control is necessary to help further reduce the burden of smoking, especially in medical schools.

## References

[1] World Health Organization (WHO) Website. Why is tobacco a public health priority? Available online at: http://www.who.int/tobacco/health_priority/en/index.html [Accessed: 1 June 2012].

[2] World Health Organization (WHO) Website. The Role of Health Professionals in Tobacco Control. Available online at: http://www.paho.org/English/DD/PUB/bookletfinal_20april.pdf [Accessed: 1 June 2012].

[3] Smith DR, Leggat PA. Smoking among Healthcare Professionals. Sydney: Darlington Press; 2011. 116pp.

[4] Chapman S Doctors who smoke. BMJ. 1995;311:142–3 10.1136/bmj.311.6998.142 7613415 PMC2550216

[5] Garfinkel L Cigarette smoking among physicians and other health professionals, 1959–1972. CA Cancer J Clin. 1976;26:373–5 10.3322/canjclin.26.6.373 825202

[6] Agency for Healthcare Research and Quality Website. Treating Tobacco Use and Dependence: 2008 Update. Available online at: http://www.ahrq.gov/path/tobacco.htm [Accessed: 14 August 2012].

[7] Davis RM When doctors smoke. Tob Control. 1993;2:187–8 10.1136/tc.2.3.187

[8] Adriaanse H, van Reek J Physicians’ smoking and its exemplary effect. Scand J Prim Health Care. 1989;7:193–6 10.3109/02813438909088663 2626609

[9] Smith DR, Leggat PA An international review of tobacco smoking in the medical profession: 1974–2004. BMC Public Health. 2007;7:115 10.1186/1471-2458-7-115 17578582 PMC1906758

[10] Kanayama H, Sato K, Mori T, Hirai T, Umemura T, Tamura T, Work-related allergy in medical doctors: atopy, exposure to domestic animals, eczema induced by common chemicals and membership of the surgical profession as potential risk factors. Int Arch Occup Environ Health. 2012;85:455–66 10.1007/s00420-011-0682-z 21853315 PMC3334482

[11] US National Library of Medicine (NLM). PubMed website. Available online at: http://www.ncbi.nlm.nih.gov/pubmed/ [Accessed: 1 June 2012].

[12] Japana Centra Revuo Medicina. Ichushi Website. Available online at: http://www.jamas.or.jp/ [Accessed: 1 June 2012].

[13] Sato H, Araki S, Yokoyama K Influence of monopoly privatization and market liberalization on smoking prevalence in Japan: trends of smoking prevalence in Japan in 1975–1995. Addiction. 2000;95:1079–88 10.1046/j.1360-0443.2000.95710799.x 10962772

[14] Nishizumi M, Kuratsune M A survey of smoking habits of physicians in Western Japan. Nihon Koshu Eisei Zasshi. 1967;14:1273–94

[15] Kono S, Ikeda M, Tokudome S, Nishizumi M, Kuratsune M Smoking and mortalities from cancer, coronary heart-disease and stroke in male Japanese physicians. J Cancer Res Clin Oncol. 1985;110:161–4 10.1007/BF00402732 4044630 PMC12253684

[16] Kono S, Ikeda M, Tokudome S, Nishizumi M, Kuratsune M Cigarette-smoking, alcohol and cancer mortality—a cohort study of male Japanese physicians. Jpn J Cancer Res. 1987;78:1323–8 3123436

[17] Wada K, Yoshikawa T, Goto T, Hirai A, Matsushima E, Nakashima Y, Lifestyle habits among physicians working in hospitals in Japan. Nippon Ishikai Zasshi. 2010;139:1894–9 (in Japanese)

[18] Wu SY, Li HY, Wang XR, Yang SJ, Qiu H A comparison of the effect of work stress on burnout and quality of life between female nurses and female doctors. Arch Environ Occup Health. 2011;66:193–200 10.1080/19338244.2010.539639 22014191

[19] Adriaanse H, Van Reek J, Van Zutphen WM Worldwide smoking habits of physicians; a survey of 100 studies of tobacco use among physicians in 31 countries in 1951–1985. Ned Tijdschr Geneeskd. 1986;130:2224–9 (in Dutch) 3808102

[20] Smith DR The historical decline of tobacco smoking among United States physicians: 1949–1984. Tob Induc Dis. 2008;4:9 10.1186/1617-9625-4-9 18822167 PMC2556033

[21] Doll R, Peto R, Boreham J, Sutherland I Mortality in relation to smoking: 50 years’ observations on male British doctors. BMJ. 2004;328:1519 10.1136/bmj.38142.554479.AE 15213107 PMC437139

[22] Smith DR, Leggat PA The historical decline of tobacco smoking among Australian physicians: 1964–1997. Tob Induc Dis. 2008;4:13 10.1186/1617-9625-4-13 19114012 PMC2646683

[23] Ponniah S, Bloomfield A An update on tobacco smoking among New Zealand health care workers, the current picture, 2006. N Z Med J. 2008;121:103–5 18425163

[24] Sato H, Araki S Yields and daily consumption of cigarettes in Japan in 1969–1996. J Epidemiol. 2000;10:7–15 10.2188/jea.10.7 10695255

[25] Sato H Policy and politics of smoking control in Japan. Soc Sci Med. 1999;49:581–600 10.1016/S0277-9536(99)00087-8 10452415

[26] World Health Organization (WHO) Website. Gender, Health, Tobacco and Equity. Available online at: http://www.who.int/tobacco/publications/gender/gender_tobacco_2010.pdf [Accessed: 1 June 2012].

[27] Tamakoshi A, Kawado M, Ozasa K, Tamakoshi K, Lin Y, Yagyu K, Impact of smoking and other lifestyle factors on life expectancy among japanese: findings from the Japan Collaborative Cohort (JACC) Study. J Epidemiol. 2010;20:370–6 10.2188/jea.JE20100017 20631456 PMC3900831

[28] Ozasa K, Katanoda K, Tamakoshi A, Sato H, Tajima K, Suzuki T, Reduced life expectancy due to smoking in large-scale cohort studies in Japan. J Epidemiol. 2008;18:111–8 10.2188/jea.JE2007416 18480591 PMC4771605

[29] Katanoda K, Marugame T, Saika K, Satoh H, Tajima K, Suzuki T, Population attributable fraction of mortality associated with tobacco smoking in Japan: a pooled analysis of three large-scale cohort studies. J Epidemiol. 2008;18:251–64 10.2188/jea.JE2007429 19075498 PMC4771610

[30] Katanoda K, Levy DT, Nakamura M, Hagimoto A, Oshima A Modeling the effect of disseminating brief intervention for smoking cessation at medical facilities in Japan: a simulation study. Cancer Causes Control. 2012;23:929–39 10.1007/s10552-012-9964-3 22527171

[31] Smith DR, L’Abbate N, Lorusso A Tobacco smoking among Italian physicians and the role of occupational medicine. Med Lav. 2008;99:3–7 18254534

[32] Hayasaki T Smoking in university hospitals and their affiliated hospitals in Japan. Int Med J. 2004;11:263–6

[33] Tobacco Control Japan Website. Current Status of Clean-Air Medical Universities in Japan. Available online at: http://www.tobacco-control.jp/medical-school-hosp-table.htm [Accessed: 14 August 2012].

[34] Saika K, Sobue T, Katanoda K, Tajima K, Nakamura M, Hamajima N, Smoking behavior and attitudes toward smoking cessation among members of the Japanese Cancer Association in 2004 and 2006. Cancer Sci. 2008;99:824–7 10.1111/j.1349-7006.2008.00736.x 18307540 PMC11159256

[35] Smith DR, Takahashi K Too many Japanese university students are still smoking tobacco. Tob Induc Dis. 2008;4:10 10.1186/1617-9625-4-10 19017385 PMC2596093

[36] Anonymous. Campus-wide smoking bans being adopted by less than half of all Japanese medical schools and hospitals (authors’ translation). Mainichi Shimbun Newspaper. February 11, 2007 (in Japanese).

[37] Smith DR, Leggat PA An international review of tobacco smoking among dental students in 19 countries. Int Dent J. 2007;57:452–8 18265779 10.1111/j.1875-595x.2007.tb00149.x

[38] Smith DR, Leggat PA A comparison of tobacco smoking among dentists in 15 countries. Int Dent J. 2006;56:283–8 17069071 10.1111/j.1875-595x.2006.tb00102.x

[39] Ojima M, Hanioka T, Tanaka H Necessity and readiness for smoking cessation intervention in dental clinics in Japan. J Epidemiol. 2012;22:57–63 10.2188/jea.JE20110038 22156286 PMC3798581

[40] Saito A, Nishina M, Murai K, Mizuno A, Ueshima F, Makiishi T, Health professional’s perceptions of and potential barriers to smoking cessation care: a survey study at a dental school hospital in Japan. BMC Res Notes. 2010;3:329 10.1186/1756-0500-3-329 21138553 PMC3016266

[41] Wakai K, Naito M, Naito T, Nakagaki H, Umemura O, Yokota M, Longitudinal Evaluation of Multi-phasic, Odontological and Nutritional Associations in Dentists (LEMONADE Study): study design and profile of nationwide cohort participants at baseline. J Epidemiol. 2009;19:72–80 10.2188/jea.JE20070458 19265274 PMC3924117

[42] Takeuchi Y, Morita E, Naito M, Hamajima N Smoking rates and attitudes to smoking among medical students: a 2009 survey at the Nagoya University School of Medicine. Nagoya J Med Sci. 2010;72:151–9 20942270 PMC11259150

[43] Tamaki T, Kaneita Y, Ohida T, Yokoyama E, Osaki Y, Kanda H, Prevalence of and factors associated with smoking among Japanese medical students. J Epidemiol. 2010;20:339–45 10.2188/jea.JE20090127 20530918 PMC3900795

[44] Smith DR, Leggat PA An international review of tobacco smoking among medical students. J Postgrad Med. 2007;53:55–62 10.4103/0022-3859.30333 17244976

[45] Kiyohara K, Kawamura T, Itani Y, Matsumoto Y, Takahashi Y Changes in teachers' smoking behaviour following enforcement of a total smoke-free school policy. Public Health. 2012;126:678–81 10.1016/j.puhe.2012.02.011 22621804

[46] World Health Organization (WHO) Website. WHO Report on the Global Tobacco Epidemic, 2011: Warning about the dangers of tobacco. Available online at: http://whqlibdoc.who.int/publications/2011/9789240687813_eng.pdf [Accessed: 1 June 2012].

[47] Smith DR, Beh EJ Hirayama, passive smoking and lung cancer: 30 years on and the numbers still don't lie. Public Health. 2011;125:179–81 10.1016/j.puhe.2011.02.008 21661135

[48] Hirayama T Non-smoking wives of heavy smokers have a higher risk of lung cancer: a study from Japan. Br Med J (Clin Res Ed). 1981;282:183–5 10.1136/bmj.282.6259.183 6779940 PMC1503989

[49] Personal communication with Judith Mackay, 20 November 2010.

[50] Japan Medical Association (JMA) Website. Declaration on the Promotion of Non-Smoking (in Japanese). Available online at: http://www.med.or.jp/people/nonsmoking/declare.html [Accessed: 12 June 2012].

[51] Japanese Respiratory Society Website. Regulations for Board Certified Physicians (in Japanese). Available online at: http://www.jrs.or.jp/home/modules/institution/index.php?content_id=1 [Accessed: 12 June 2012].

[52] Kaetsu A, Fukushima T, Moriyama M, Shigematsu T Smoking behavior and related lifestyle variables among physicians in Fukuoka, Japan: a cross sectional study. J Epidemiol. 2002;12:199–207 10.2188/jea.12.199 12164321 PMC10499475

[53] Kawane H Tobacco Smoking in Japan. CMAJ. 1986;135:971 3756735 PMC1491289

[54] Kawane H Smoking and older chest physicians. Chest. 1991;99:526 10.1378/chest.99.2.526a 1750861

[55] Kawane H The prevalence of smoking among physicians in Japan. Am J Public Health. 1993;83:1640 10.2105/AJPH.83.11.1640 8238697 PMC1694897

[56] Kawane H Smoking among Japanese physicians. JAMA. 2001;286:917 10.1001/jama.286.8.917 11509047

[57] Kaetsu A, Fukushima T, Moriyama M, Shigematsu T Change of the smoking behavior and related lifestyle variables among physicians in Fukuoka, Japan: a longitudinal study. J Epidemiol. 2002;12:208–16 10.2188/jea.12.208 12164322 PMC10499472

[58] Miwa K, Fujita M, Miyagi Y, Inoue H, Sasayama S Is smoking behaviour in patients with coronary heart disease influenced by whether their attending physician smokes? Tob Control. 1995;4:236–8 10.1136/tc.4.3.236

[59] Audet B When it comes to smoking, Japanese MDs do not set a good example for their patients. CMAJ. 1994;150:1673–4 8174037 PMC1336982

[60] Kawane H, Soejima R Smoking among doctors in a medical school hospital. Kawasaki Igakukai Shi. 1996;22:211–6

[61] Kawakami M, Nakamura S, Fumimoto H, Takizawa J, Baba M Relation between smoking status of physicians and their enthusiasm to offer smoking cessation advice. Intern Med. 1997;36:162–5 10.2169/internalmedicine.36.162 9144005

[62] Kawahara K, Ohida T, Osaki Y, Mochizuki Y, Minowa M, Yamaguchi N, Study of the smoking behavior of medical doctors in Fukui, Japan and their antismoking measures. J Epidemiol. 2000;10:157–62 10.2188/jea.10.157 10860299

[63] Ohida T, Sakurai H, Mochizuki Y, Kamal AM, Takemura S, Minowa M, Smoking prevalence and attitudes toward smoking among Japanese physicians. JAMA. 2001;285:2643–8 10.1001/jama.285.20.2643 11368741

[64] Ohida T, Kamal AM, Takemura S, Sakurai H Letter to the Editor: In reply. JAMA. 2001;286:917

[65] Kaneita Y, Sakurai H, Tsuchiya T, Ohida T Changes in smoking prevalence and attitudes to smoking among Japanese physicians between 2000 and 2004. Public Health. 2008;122:882–90 10.1016/j.puhe.2007.12.009 18561965

[66] Wada K, Sakata Y, Theriault G, Narai R, Yoshino Y, Tanaka K, Associations of excessive sleepiness on duty with sleeping hours and number of days of overnight work among medical residents in Japan. J Occup Health. 2007;49:523–7 10.1539/joh.49.523 18075214

[67] Kai T, Maki T, Takahashi S, Warner DO Perioperative tobacco use interventions in Japan: a survey of thoracic surgeons and anaesthesiologists. Br J Anaesth. 2008;100:404–10 10.1093/bja/aem400 18234680

[68] Kaneita Y, Uchida T, Ohida T Epidemiological study of smoking among Japanese physicians. Prev Med. 2010;51:164–7 10.1016/j.ypmed.2010.04.015 20438746

[69] Wada K, Yoshikawa T, Goto T, Hirai A, Matsushima E, Nakashima Y, Lifestyle habits among physicians working at hospitals in Japan. Jpn Med Assoc J. 2011;54:318–24

[70] Yokota B Smoking among physicians in Osaka prefecture. Osakafu Ishikai News. 1980;May:1247–8 (in Japanese).

[71] Nishizumi M The change of smoking and drinking habits among physicians. Kousei no Shihyo. 1986;33:33–9 (in Japanese).

[72] Yokota B Actual smoking status among physicians and teachers. Nippon Iji Shinpo. 1988;April:95–7 (in Japanese).

[73] Kawane H, Soejima R, Yagi S, Okimoto N, Umeki S, Kishimoto T, Smoking among physicians of a medical school hospital. Kawasaki Igakukai Shi. 1989;15:351–7 (in Japanese)

[74] Nomura Y, Kayama Y, Matsui K, Katsuro H, Tanaka S Smoking status among medical workers at Yokosuka City Hospital. Kanagawa Igakukai Zasshi. 1991;18:19–22 (in Japanese)

[75] Igarashi S, Muroya S Smoking condition of members of the Medical Association of Toyama. Toyama Kenritsu Chuo Byoin Igaku Zasshi. 1989;1012:18–21 (in Japanese)

[76] Kawane H, Soejima R Smoking among members of the Japan Society of Chest Diseases. Nihon Kyobu Shikkan Gakkai Zasshi. 1991;29:182–5 (in Japanese) 2033892

[77] Kobayashi J, Kitamura S Results of a questionnaire about smoking distributed at the 36th Annual Meeting of the Japan Society of Chest Diseases. Nihon Kyobu Shikkan Gakkai Zasshi. 1997;35:863–6 (in Japanese) 9366160

[78] Kano M, So K, Iwashita T, Kawakami N, Shimizu H Smoking prevalence and degree of tobacco dependence of physicians. Nihon Koshu Eisei Zasshi. 1999;46:658–63 (in Japanese) 10496035

[79] Ohida T, Kawahara K, Osaki Y, Sone T, Ishii T, Fujimori T, Smoking behaviors among medical doctors in Fukui, Japan. Nippon Eiseigaku Zasshi. 2000;55:559–65 (in Japanese) 10.1265/jjh.55.559 11215142

[80] Kawakami M, Nakamura S, Kaneko H, Takizawa J, Baba M, Mikami M Difference in smoking status and smoking cessation intervention between clinic doctors and general-hospital doctors. Kikanshi Gaku. 1998;20:467–71 (in Japanese)

[81] Sakurai H, Ohida T Smoking behaviors and attitude to smoking among members of the Japanese Medical Association. Nippon Ishikai Zasshi. 2000;124:725–36 (in Japanese)

[82] Takiguchi Y, Kurosu K, Kasahara Y, Tanabe N, Tatsumi K, Kuriyama T Smoking and attitude toward smoking of respiratory care specialists. Nihon Kokyu Kanri Gakkaishi. 2004;13:490–5 (in Japanese)

[83] Kaneita Y, Ohida T Smoking behavior and attitude to smoking among members of the Japan Medical Association in 2004. Nippon Ishikai Zasshi. 2005;133:505–17 (in Japanese)

[84] Kaneita Y, Ohida T, Uchida T Prevalence and correlates of smoking among Japanese physicians: result from the 2008 survey on the smoking activities of Japan Medical Association members. Nippon Ishikai Zasshi. 2009;138:770–7 (in Japanese)

[85] Miyahara H, Yoshino K, Asai M, Ikeda H, Sakuraba M, Shirasuna K, Questionnaire survey of members of the Japan Society for Head and Neck Cancer concerning smoking habits. Toukeibugan. 2009;35:250–6 (in Japanese)

